# Impedance Sensing and Characterization of Single-Cell Migration in Channels with Selective Protein Coating

**DOI:** 10.3390/bios16050290

**Published:** 2026-05-16

**Authors:** Xiao Hong, Stella W. Pang

**Affiliations:** 1 Department of Electrical Engineering, City University of Hong Kong, Hong Kong, China; 2 Centre for Biosystems, Neuroscience, and Nanotechnology, City University of Hong Kong, Hong Kong, China; 3 State Key Laboratory of Terahertz and Millimeter Waves, City University of Hong Kong, Hong Kong, China

**Keywords:** impedance sensing, single-cell migration, cell identification, MC3T3 and NP460 cells

## Abstract

Understanding cell migration is essential not only for fundamental biology but also for the development of targeted disease therapies. Traditional *in vitro* cell migration assays typically rely on optical microscopy to capture cell movements and subsequent image-based tracking to quantify cell migration characteristics, which often involve substantial experimental workload and analytical complexity. Therefore, there is a need for an automated and streamlined approach to monitor and analyze cell movements. In this work, a microfabricated impedance sensor integrating electrode pairs and selectively protein-coated channels was developed for real-time monitoring of single-cell migration. The optimized electrode dimensions with 10 μm width and 10 μm gap enabled sensitive detection of impedance magnitude increase induced by individual cells. The impedance magnitude changes were correlated with the cell coverage area on electrodes, allowing continuous tracking of single-mouse osteoblast MC3T3 cell movement across the electrode pair. Distinct impedance responses of signal duration and magnitude were observed under different surface coatings, revealing the influence of microenvironmental chemistry on cell motility and adhesion. Furthermore, comparative impedance profiling of MC3T3 and nasopharyngeal epithelial NP460 cells demonstrated that MC3T3 cells produced larger changes in impedance real part and phase due to larger spreading area and larger number of focal adhesions, whereas NP460 cells showed shorter impedance signal change durations, consistent with faster cell migration. These electrical signatures collectively captured intrinsic differences in cell morphology, adhesion, and motility. The developed impedance sensor provides a label-free approach for single-cell migration characterization and can be potentially applied to cell identification.

## 1. Introduction

Cell migration is essential for physiological processes such as immune response and wound healing [1,2], and its abnormal regulation is closely associated with disease progression [3]. Therefore, a deep understanding and quantification of cell migration is important for biological research and clinical diagnosis. Among the widely used techniques, wound-healing assay studies two-dimensional cell migration. It involves scratching a cell monolayer and monitoring the gap closure to evaluate cell motility. Despite being straightforward, the scratching process inevitably causes mechanical damage to cells, which can hinder migration behaviors and compromise the results’ accuracy [4]. The transwell assay represents another approach, where cells migrate through a porous membrane. However, this technique requires multiple manual steps, making it highly labor-intensive and time-consuming [5]. Recently, three-dimensional (3D) cell culture platforms have been developed to closely mimic *in vivo* microenvironments using micro- and nanofabricated substrates [6,7,8,9,10]. While these systems offer improved physiological relevance, analyzing cell migration typically relies on microscopy-based observation and image-based analysis to quantify cell movements, leading to substantial analytical complexity. Therefore, there is a demand for a streamlined strategy to monitor cell migration with reduced analytical effort.

Over the past few decades, electrical cell–substrate impedance sensing (ECIS) has attracted attention as a non-invasive and label-free technique for monitoring cellular behaviors. In ECIS, microfabricated gold electrodes are deposited at the bottom of the culture chambers, and a weak alternating current (AC) voltage is applied to measure the impedance between the electrode and the surrounding medium. As the cells attach, migrate, and spread on the electrode surface, they modulate the current pathways and consequently alter the measured impedance [11,12,13]. While ECIS has been well-established for investigating collective cellular processes [14,15,16,17,18,19,20,21,22,23,24], its application at the single-cell level is still limited and challenging. The relatively large electrode dimensions and limited spatial resolution in conventional ECIS setups make it difficult to resolve the subtle impedance variations induced by individual cells. Moreover, insufficient cell adhesion on the electrode surface can further attenuate the signal amplitude. Weak adhesion results in a larger gap between the cell membrane and the electrode surface, creating a larger current leakage pathway that compromises measurement sensitivity [25]. To achieve single-cell detection sensitivity, extensive efforts have been made to miniaturize electrode dimensions to the cellular scale and modify the electrode surface chemistry to enhance cell–electrode coupling [25,26,27,28]. However, most of these studies focused on static or adhered single cells rather than capturing dynamic cellular behaviors. There remains a lack of research on real-time impedance tracking of cell migration at the single-cell level.

Monitoring cell migration through impedance sensing is essential not only for understanding cell motility but also for uncovering intrinsic biophysical differences between cell types. Distinct cell populations often display characteristic electrical behaviors arising from differences in cell size, morphology, and motility [29,30]. These features collectively constitute a unique impedance fingerprint for each cell type, forming the theoretical basis for label-free cell identification using ECIS-based techniques. By extracting multiple parameters from the measured impedance signals, a multidimensional electrical profile can be constructed for each cell, enabling quantitative distinction between different cell types without the need for fluorescence labeling or high-end microscopy.

In this work, a novel impedance sensor based on ECIS was developed for real-time monitoring of single-cell migration [31]. The device consisted of coplanar electrode pairs with optimized dimensions in SU8 channels. The channels were selectively coated with extracellular matrix (ECM) proteins on the bottom and sidewalls, while the top of the channels remained uncoated. Since ECM proteins such as fibronectin (FN) could promote cell attachment and adhesion, cells preferentially resided on the coated bottoms and sidewalls of the channels and rarely translocated to the top surface. The coating confined cells to migrate in the channels to contact the electrodes on the bottom, and not to escape to the top of the channels. This sensor successfully detected individual mouse osteoblast MC3T3-E1 cells passing through the electrode pair within the channel. Analysis of the impedance signal duration and magnitude revealed the influence of different protein coatings on MC3T3 cell migration and adhesion dynamics, which was consistent with optical observations. In addition, the migratory behaviors of MC3T3 cells and nasopharyngeal epithelial NP460 cells were compared. Distinct impedance characteristics were observed between these two cell types. These findings underscore the usefulness of the developed sensor for label-free cell identification based on impedance-derived parameters. To the best of our knowledge, this study represents the first demonstration of continuous impedance profiling to track single-cell migration within protein functionalized channels. The proposed sensor offers a label-free approach for characterizing single-cell migration and establishes a foundation for potential cell identification.

## 2. Materials and Methods

### 2.1. Design and Fabrication Technology for Impedance Sensor

**Figure 1:**
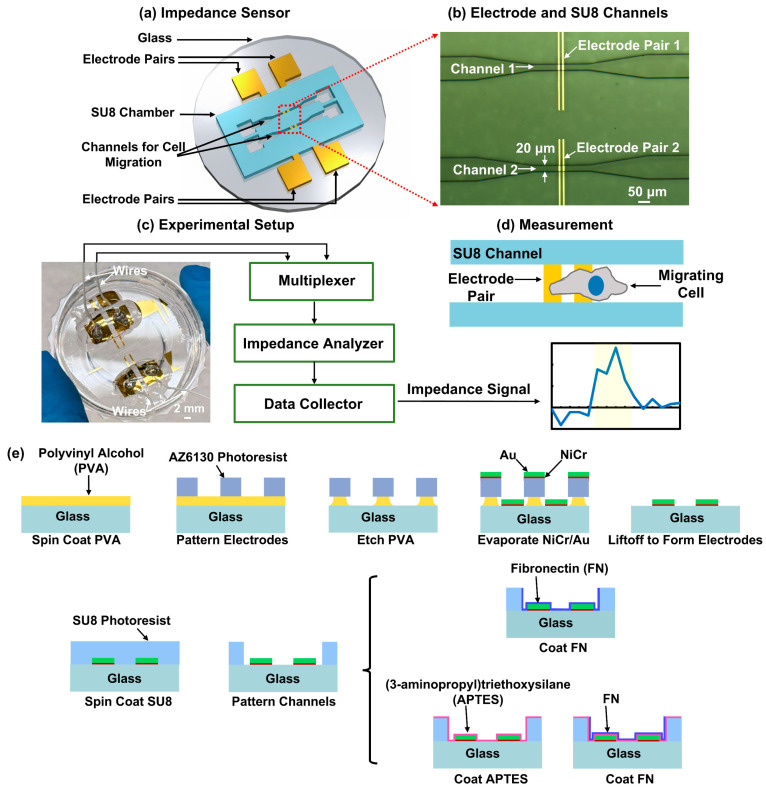
Design and fabrication of impedance sensor. (**a**) Schematic of impedance sensor. (**b**) Micrograph of 20 μm-wide SU8 channels on top of electrode pairs. Electrode pairs had width of 10 μm and gap of 10 μm. (**c**) Experimental setup for impedance measurement of single migrating cells. (**d**) Impedance signal over time from single-cell migrating passed across electrode pair. (**e**) Schematics of fabrication technology for impedance sensor with FN or APTES + FN coating in channel bottom and sidewalls.

An impedance sensor employing electrode pairs with overlying SU8 channels was designed in this work, as shown in Figure 1a. The device comprised two electrode pairs patterned on a glass substrate. An SU8 microfluidic chamber containing two channels for cell migration was aligned on top of the electrodes. An optical micrograph of the fabricated NiCr/Au electrodes and SU-8 channels is shown in Figure 1b. The electrodes consisted of a 10 nm thick NiCr adhesion layer and a 120 nm thick Au layer, with both the electrode width and interelectrode gap at 10 μm. The SU8 channels were 20 μm wide and 15 μm high. Experimental setup for impedance measurement of individual migrating cells is shown in Figure 1c. Two pairs of electrodes were connected to the multiplexer and impedance analyzer for collection of impedance over time. As individual cells migrated through the electrodes, impedance signal increased and recorded for further analysis, as shown in Figure 1d.

Figure 1e illustrates the fabrication technology for impedance sensors with selective protein coating. Electrodes were formed by a liftoff process with water-soluble polyvinyl alcohol (PVA, Invitrogen, Waltham, MA, USA) as a sacrifice layer. Since PVA could be dissolved in water, the fluidic platforms remained free of organic residues that typically persist after the conventional liftoff process, using organic solvents to remove photoresist. The use of PVA minimized organic residues, which is beneficial for cell viability during long-term live cell measurements. Firstly, the glass substrate was cleaned, followed by an O_2_ plasma treatment to enhance surface hydrophilicity. A 4% (weight by volume) PVA solution was spin-coated onto the glass substrate and baked at 105 °C for 2 min. Subsequently, the electrode pattern was defined on the PVA film by photolithography using AZ6130 positive photoresist (Merck KGaA, Darmstadt, Germany), followed by reactive ion etching (RIE) the PVA to generate an undercut profile. The RIE condition was 20 sccm O_2_, 40 mTorr, and 100 W radio frequency (rf) power for 15 min. A 10 nm NiCr adhesion layer and a 120 nm Au layer were then deposited by thermal evaporation (DV-502B, Denton Vacuum, Moorestown, NJ, USA). The sacrificial PVA layer was subsequently dissolved in DI water and the NiCr/Au electrodes were formed on the glass substrate. Then, SU8 channels were aligned and patterned on top of the electrodes using photolithography. External wires were soldered onto the electrodes and encapsulated with polydimethylsiloxane (PDMS, Sylgard 184, Dow, Midland, MI, USA). The glass substrate with the electrodes and channels was then bonded to a cell culture dish using PDMS and baked at 80 °C for 8 h.

To improve device biocompatibility and cell adhesion in channels, selective protein coating was applied to the channel bottom and sidewalls under two conditions: FN alone and a sequential (3-aminopropyl)triethoxysilane (APTES) plus FN (APTES + FN) treatment. Before coating, the device was exposed to an O_2_ plasma with a condition of 20 sccm, 100 mTorr, and 75 W rf power for 1 min. For FN coating, a 60 µg/mL FN solution (Sigma-Aldrich, St. Louis, MO, USA) was introduced through the SU8 chamber inlet with a flat PDMS pad covering the top surface so that coating was on the sidewalls and bottom of the channels, and not on the top. The device was incubated at 4 °C for 3 h. For APTES + FN coating, the device was first immersed in a 10% (volume by volume) APTES (Sigma-Aldrich, St. Louis, MO, USA) in ethanol at 25 °C for 15 min, allowing APTES to coat all surfaces. Next, FN was selectively coated on the channel bottom and sidewalls, and not on the top surfaces of the platforms. Excess FN was removed by rinsing with DI water.

### 2.2. Fabrication Technology for Microfluidic Device with Single Electrode Pair

The fabrication technology for the microfluidic device used for floating cell experiment is illustrated in Supplementary Figure S1a. The microfluidic device consisted of a coplanar electrode pair beneath a PDMS channel. The electrode pair with 10 nm NiCr adhesion layer and a 120 nm Au layer was fabricated using the same technology as described above. The PDMS channel was replicated from a saline-coated silicon (Si) stamp fabricated by photolithography and deep RIE. A PDMS prepolymer mixture of base and curing agent at 10:1 weight ratio was poured onto the Si stamp and baked at 80 °C for 8 h. After curing, the PDMS layer was peeled off, and inlet and outlet ports were punched at two ends of the PDMS channel. The PDMS channel was then aligned with the electrode pair and bonded to the glass substrate via an O_2_ plasma activation. External wires were soldered onto the electrodes and encapsulated with PDMS. Finally, the glass substrate containing the electrodes and the PDMS channel was bonded to a cell culture dish using PDMS and baked at 80 °C for 8 h. The micrograph of the assembled device is shown in Supplementary Figure S1b.

### 2.3. Cell Culture and Seeding

Mouse osteoblast MC3T3-E1 cells (American type culture collection, CRL-2594, Manassas, VA, USA) and immortalized nasopharyngeal epithelial NP460 cells (S. W. Tsao, the University of Hong Kong, China) were used in this study. MC3T3 cells were maintained in a high-glucose Dulbecco’s modified eagle medium (DMEM, Invitrogen, USA) with 10% fetal bovine serum (FBS, Gibco, Waltham, MA, USA), 1% antibiotic antimycotic (Gibco, Waltham, MA, USA), and 1% alanyl-L-glutamine (Gibco, Waltham, MA, USA). NP460 cells were cultured in a mixed medium comprising EpiLife medium (Gibco, Waltham, MA, USA) with 1% EpiLife defined growth supplement (Gibco, Waltham, MA, USA), and defined keratinocyte serum-free medium (Gibco, Waltham, MA, USA) with 0.2% defined keratinocyte growth supplement (Gibco, Waltham, MA, USA). The NP460 cell culture medium was further supplemented with 1% antibiotic antimycotic (Gibco, Waltham, MA, USA). All cells were incubated at 37 °C and 5% CO_2_.

### 2.4. Impedance Measurement and Time-Lapse Imaging

For migrating cell experiments, 5 × 10^4^ MC3T3 cells or 7 × 10^4^ NP460 cells in 2 mL of CO_2_ medium were evenly seeded onto the device. The CO_2_ medium consisted of CO_2_ independent medium (Gibco, Waltham, MA, USA) supplemented with 10% FBS (Gibco, Waltham, MA, USA), 1% antibiotic antimycotic (Gibco, Waltham, MA, USA), and 1% GlutaMAX (Gibco, Waltham, MA, USA). After 2 h initial cell attachment, the impedance measurement and time-lapse imaging commenced simultaneously. An impedance analyzer (Reference 600+ Potentiostats, Gamry, Warminster, PA, USA) coupled with an electrochemical multiplexer (IMX8, Gamry, Warminster, PA, USA) was used to apply the excitation electric field and acquire impedance signals from two electrode pairs separately. Measurements were performed at 100 kHz with a 30 mV AC voltage amplitude. The choice of measurement frequency is based on the relative contributions of capacitive and resistive elements in the cell–electrode system. At very low frequencies, the electrical double layer at the electrode–electrolyte interface presents a high and unstable capacitive impedance, which dominates the total impedance signal and introduces noise arising from the fluctuations at the electrode–electrolyte interface. At very high frequencies, the double layer capacitance becomes negligible, but the contribution of cell capacitive elements also decreased, resulting in lower sensitivity. As a result, the mid frequency range of 10 to 100 kHz was found to be more sensitive for ECIS-based cell migration measurements [32]. In this study, the impedance magnitude of single-cell passage across electrodes was evaluated at 10, 33, and 100 kHz, as shown in Supplementary Figure S2. As the frequency increased, the noise from electrode–electrolyte interface progressively diminished. Among these frequencies, 100 kHz provided the lowest noise level and was, therefore, selected as the frequency for impedance evaluations. Electrical readings including impedance magnitude, phase, and real and imaginary parts were collected every 10 min for 24 h.

Cell positions in the SU8 channel were recorded by time-lapse imaging using an upright microscope (Eclipse NI-U, Nikon, Tokyo, Japan) at 10 min intervals over 24 h. The microscope was connected to a cell culture incubator to maintain chamber humidity and temperature. For floating cell experiments, MC3T3 cells suspended in CO_2_ medium were continuously injected into the microfluidic device and the changes in impedance were measured by the same impedance analyzer as described above. Time-lapse imaging of MC3T3 cells passing over electrodes were acquired using the Nikon upright microscope.

### 2.5. Cell Staining and Immunofluorescence Imaging

After 26 h cell culture, MC3T3 and NP460 cells were fixed with 4% paraformaldehyde (PFA, Sigma-Aldrich, St. Louis, MO, USA) for 15 min. For immunofluorescence imaging, fixed cells were permeabilized with 0.1% Triton X-100 solution (ThermoFisher, Waltham, MA, USA) for 10 min and blocked with 1% bovine serum albumin (ThermoFisher, USA) for 30 min. Cells were then stained with FAK100 actin cytoskeleton/focal adhesion (FA) staining kit (Merck, Rahway, NJ, USA) using standard protocol. The immunofluorescence images were captured using a confocal microscope (STELLARIS 8, Leica, Wetzlar, Germany).

### 2.6. Scanning Electron Microscopy (SEM)

To visualize the morphology of MC3T3 and NP460 cells within channels, cells were fixed with 4% PFA (Sigma-Aldrich, St. Louis, MO, USA) for 15 min after 26 h of culture. They were then immersed in 1× phosphate-buffered saline (Gibco, USA) for 5 min, DI water for 10 min, and dehydrated through a graded ethanol series at 30%, 50%, 70%, 90%, 95%, and 100%, each for 5 min. The cells were subsequently dried using a critical point dryer (EM CPD300, Leica, Wetzlar, Germany) and coated with a thin layer of Au by a thin film coater (Q150 coater, Quorum Technologies, East Sussex, UK). Images of the fixed cells were taken by a scanning electron microscope (SU5000, Hitachi, Tokyo, Japan).

### 2.7. Impedance Analysis

For migrating cell experiments, the impedance magnitude Z(t) was recorded every 10 min over a 24 h period. When no cells were on the electrodes, the measured Z(t) was defined as the background impedance, Z_background_(t). During the 24 h measurement, gradual evaporation of the culture medium led to increased ion concentration in the medium, resulting in a slow decrease in the background impedance. To correct this baseline drift, the background impedance was modeled as a linear function of time, Z′_background_(t) = a · t + b. The coefficients a and b were determined by fitting a first-order polynomial to the measured background impedance Z_background_(t). Using these coefficients, the fitted background impedance Z_fitted_background_(t) was reconstructed over the entire measurement duration. The change in impedance magnitude was then determined as ΔZ(t) = Z(t) − Z_fitted_background_(t). To distinguish cell induced signals from noise, the noise levels were measured and defined as one standard deviation of the impedance magnitude signal in the absence of cells, reflecting inherent electronic instrumentation and electrode–electrolyte interfacial noise [33]. Although occasional transient data points exceeded the noise level, these excursions were stochastic and non-continuous. Accordingly, a valid cell passage could be identified not only based on an increase in impedance magnitude above the noise level, but also by the temporal persistence. Impedance changes were considered as cell passages across the electrodes when the signals deviated from the baseline for continuous periods. The impedance real-part change, imaginary part change, and phase change were calculated using the same method described above.

For floating cell experiments, the impedance magnitude when no cell floated across the electrodes was defined as Z_base_. The peak impedance magnitude when a single cell floated across the electrodes was defined as Z_n_, where n represents the number of cells that floated across the electrodes. The normalized impedance magnitude was calculated as Z_n_/Z_base_ to reflect the impedance change induced by cell passage. The average impedance magnitude change was calculated using the equation below:

$$Average\;impedance\;magnitude\;change=\frac{\sum_{1}^{n}\frac{z_{n}-z_{base}}{z_{base}}}{n}\times 100\%$$

### 2.8. Cell Migration and Morphology Analysis

Cell passing speed, electrode area coverage, and cell morphology were analyzed in ImageJ software (version 1.48) integrated with a manual tracking plugin. Cell passing speed was defined as the average migration speed of a cell migrating across the area between the outer edges of electrodes. Electrode area coverage was defined as the ratio of electrode area covered by cells to total area between the outer edges of electrodes. Cell length was defined as the maximum projection length along the channel direction. Data was collected from at least three independent experiments. Statistical differences between groups were assessed by one-way analysis of variance (ANOVA) and Tukey’s *post hoc* test. Results were reported as mean ± standard error of the mean.

## 3. Results and Discussion

### 3.1. Influence of Electrode Gap and Width on Impedance Changes by Floating Cells

The sensitivity of an impedance sensor depends on the geometric sizes of electrodes, including the distance between the electrodes, known as the electrode gap, and the electrode width [32,34,35]. To systematically evaluate how electrode gap and width influence device sensitivity as single cells pass the region between the outer edges of electrodes, a series of microfluidic devices with varied electrode dimensions were fabricated. These floating cell experiments served two primary purposes. Firstly, they were designed to verify whether the impedance sensor could reliably detect individual cells. When a floating cell passed the electrode pair, it led to a transient impedance peak. The presence of this peak could confirm the sensor’s sensitivity for single-cell detection. Secondly, these experiments were used to identify the electrode dimensions that maximize impedance change due to single-cell passage.

Each microfluidic device consisted of a single coplanar electrode pair integrated at the bottom of the PDMS channel. The channel width was fixed at 20 μm to accommodate individual MC3T3 cells, and the channel height was fixed at 10 μm to ensure close contact between cells and electrodes. Two sets of electrode configurations were investigated. In the first set, to study the effect of electrode gap, the electrode width was fixed at 20 μm while the gap was varied at 10, 20, 40, and 50 μm. In the second set, to examine the effect of electrode width, the gap was fixed at 10 μm while the width was varied at 10, 15, and 20 μm. During measurements, MC3T3 cells suspended in culture medium were continuously injected into the channel and passed across the electrode pair.

**Figure 2:**
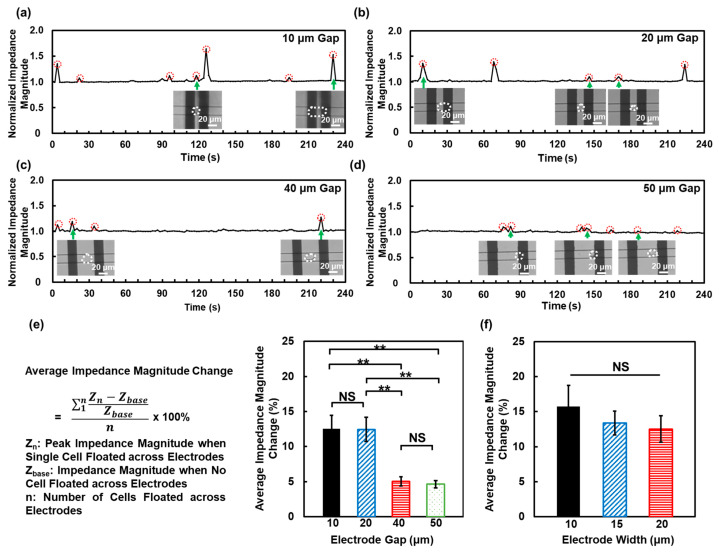
Effects of electrode gap and width on impedance magnitude changes for single floating cells. Normalized impedance magnitude increased as single floating cells passing through channels with 20 μm wide electrodes and gaps of (**a**) 10, (**b**) 20, (**c**) 40, and (**d**) 50 μm. Green arrows point to corresponding impedance points where cells in micrographs passed electrode. Red dotted circles highlight impedance peaks, each of which correspond to individual cell passage. (**e**) Average impedance magnitude change decreased with larger electrode gaps. (**f**) No significant difference in impedance magnitude changes when electrode with 10 μm gap and widths ranged from 10 to 20 μm. One-way ANOVA and Tukey’s *post hoc* test with ** *p* < 0.01 and NS—not significant.

The effect of the electrode gap was first characterized. Figure 2a–d show the normalized impedance magnitude of single MC3T3 cells passing over electrodes with gaps of 10, 20, 40, and 50 μm. When no cells were present, the normalized impedance magnitude was low due to the higher conductivity of cell culture medium. As a single MC3T3 cell passed over the electrodes, the impedance magnitude increased because the cell body partially blocked the current path. Each impedance peak circled in Figure 2a–d corresponded to an individual cell passage event, which was confirmed by optical observation and shown in the micrographs. Cells were outlined in white dashed line. The impedance peaks showed variability in their magnitude, which may result from the intrinsic heterogeneity of individual cells and their varying positions between the outer edges of the electrodes. Differences in cell sizes could influence the effective contact area between the cell membrane and the electrode surface, thereby altering the measured impedance magnitude [36,37]. In addition, the electric field generated by the coplanar electrode pair was spatially nonuniform, with the highest field intensity located near the inner edges of the electrodes due to point discharge [34]. Consequently, cells passing through different positions within the area between the outer edges of electrodes experienced varying electric field strength, leading to variations in impedance magnitude responses [38,39].

For electrode gaps of 10 and 20 μm, comparable impedance peaks were observed. However, when the gap increased to 40 and 50 μm, the peak impedance magnitude decreased markedly. The average impedance change declined with increasing electrode gap, as shown in Figure 2e. This reduction was attributed to a decrease in the effective electrode area covered by a single MC3T3 cell as the electrode gap widened. Therefore, an electrode gap of 10 μm was selected for subsequent experiments to ensure a high impedance response.

The effect of electrode width was then examined by measuring the impedance magnitude response of a single MC3T3 cell passage with electrode widths of 10, 15, and 20 μm, as shown in Supplementary Figure S3. As illustrated in Figure 2f, electrode widths did not significantly affect the impedance magnitude change, although the 10 μm electrodes showed a slightly higher mean value. Since the electrode gap was fixed at 10 μm in this set of experiments, the cells were able to effectively cover the electrode gap region where the electric field was most concentrated, leading to comparable impedance magnitude changes across different electrode widths.

### 3.2. Impedance Sensing of Single-Cell Migration Across Electrode Pairs

#### 3.2.1. Tracking Single MC3T3 Cell Migration via Impedance Magnitude Changes

Based on the optimized electrode dimensions, electrodes with 10 μm gaps and 10 μm widths were employed in the subsequent experiments. SU8 channels were patterned on top of electrodes to confine the cell movement. The channels were not covered on top to allow sufficient medium exchange and maintain cell viability. To prevent cells from migrating out of the channels and to promote cell attachment on the electrode surface, selective FN coating was applied to the channel bottom and sidewalls.

**Figure 3:**
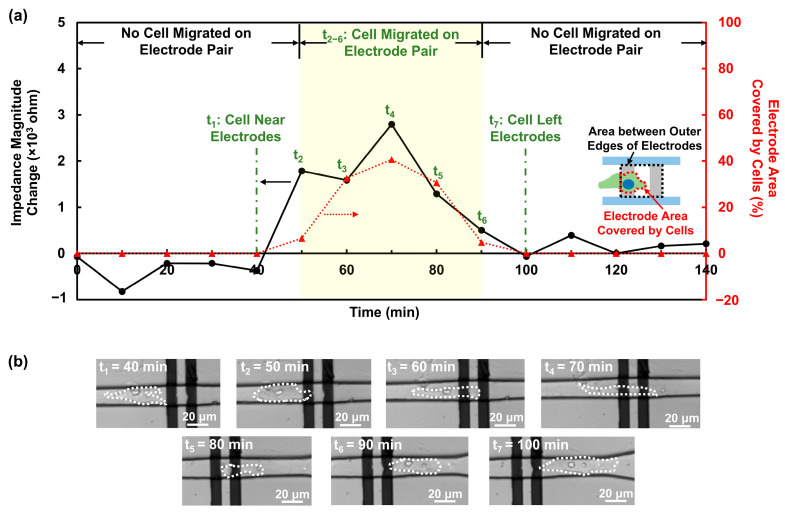
Impedance magnitude of a single MC3T3 cell migrating across a pair of electrodes with FN coating in channel. (**a**) Impedance magnitude increased when a single MC3T3 cell migrated on electrode pair during t_2_ to t_6_. Impedance magnitude change was related to electrode area coverage. (**b**) Representative time-lapse images of a single MC3T3 cell migrating across a pair of electrodes. Cell body is outlined by white dashes.

Figure 3a shows the impedance magnitude response and the electrode area coverage when a single MC3T3 cell migrated across an electrode pair with FN coating. Representative time-lapse images captured at t_1_ to t_7_ are presented in Figure 3b, with the cell bodies outlined by white dashes. From 0 to 40 min, no cell contacted the region between the outer edges of the electrodes. The optical micrograph captured at 40 min shows that the cell was near the electrode, as shown in Figure 3b. During this period, the impedance magnitude remained low. In this work, noise level was defined as one standard deviation of the impedance magnitude when no cell migrated on electrodes, which was calculated to be 360.5 ohm. At around 50 min, the MC3T3 cell first contacted the left electrode, leading to an increase in impedance magnitude above the noise level. As the cell continued to migrate forward and progressively covered a larger percentage of the area between the outer edges of the electrode from 60 to 70 min, the impedance magnitude further increased. Subsequently, from 80 to 90 min, the impedance magnitude gradually decreased as the cell moved away and covered a smaller fraction of the electrode region. After the cell left the electrode region at 100 min, the impedance signal decreased to the noise level.

These results validated that the developed impedance sensor is capable of monitoring single-cell migration in real time. The continuous increase in impedance magnitude above the noise level coincided with the interval during which the MC3T3 cell migrated on the electrode pair, establishing a direct temporal correspondence between the electrical signal and cell position. The magnitude of the impedance change scaled with the electrode area coverage, which agreed with previous reports that the cell electrode coverage modulated the impedance signal [14,40]. Greater electrode coverage by a cell generally produced stronger current constriction and, thus, a larger impedance magnitude response.

#### 3.2.2. Impedance Signal Reflected Cell Motility and Adhesion in Channels with Coatings

Cells often encounter different protein environments *in vivo* [41]. To assess the sensor’s ability to detect changes in cell migration dynamics influenced by distinct microenvironments, the channel surface was modified to regulate cell behaviors. Specifically, the salinization reagent APTES was employed to functionalize the substrate with amine groups (-NH2). This surface treatment could enhance protein immobilization through covalent coupling, thereby modulating cell adhesion and migration [42,43]. For comparison, devices were prepared with two different coatings, FN coating alone and APTES followed by FN coating, namely APTES + FN.

**Figure 4:**
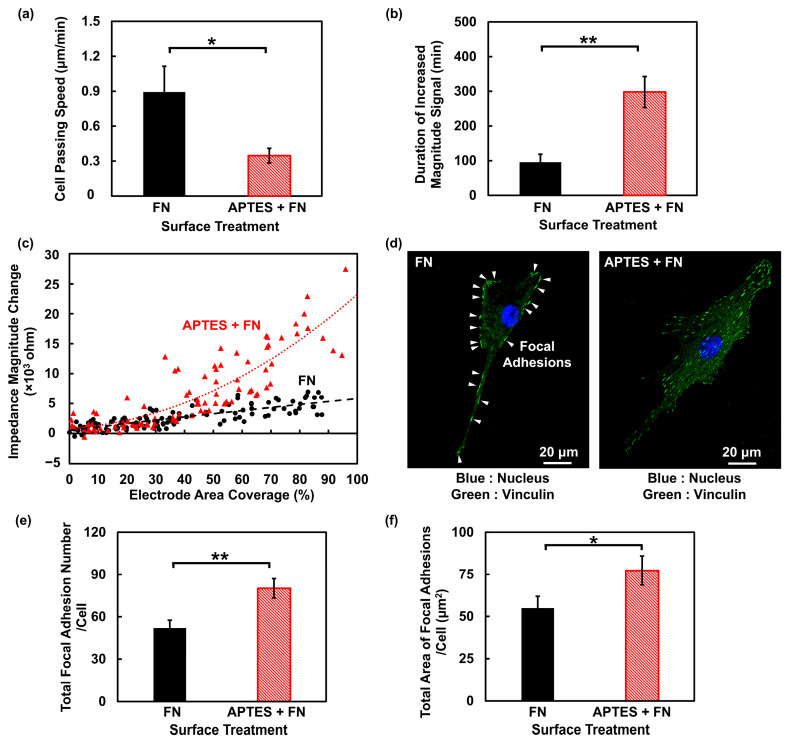
Impedance monitoring of MC3T3 cell migration across electrode pairs with FN or APTES + FN coating. (**a**) MC3T3 cells showed lower migration speed when passing electrodes with APTES + FN coating. (**b**) Duration of increased magnitude signal was longer when MC3T3 cell migrate on electrodes with APTES + FN coating. (**c**) Migration of MC3T3 cells on electrodes with APTES + FN coating led to larger impedance magnitude change. Red triangles and black dots are impedance magnitude changes when MC3T3 cells migrated on electrodes with APTES + FN and FN coating, respectively. (**d**) Immunofluorescence images of MC3T3 cells on glass with FN or APTES + FN coating. Cells were stained to observe nucleus (blue) and vinculin (green). White triangles point to FAs. (**e**) MC3T3 cells on APTES + FN-coated surfaces had larger number of focal adhesions. (**f**) MC3T3 cells on APTES + FN-coated surfaces had larger total area of focal adhesions. One-way ANOVA and Tukey’s *post hoc* test with * *p* < 0.05 and ** *p* < 0.01.

Cell migration speed across the electrodes was first quantified using time-lapse imaging. As shown in Figure 4a, MC3T3 cells showed lower passing speed on electrodes with APTES + FN coating. On FN-coated electrodes, the average MC3T3 cell passing speed was 0.89 μm/min, whereas on APTES + FN-coated electrodes, it decreased to 0.34 μm/min. In parallel, the impedance magnitude signal was analyzed to provide an electrical metric of cell migration. The duration of increased impedance magnitude signal, defined as the continuous interval during which the impedance magnitude remained above the noise level, was adopted as an indicator for cell motility. For example, in Figure 3a, this duration spanned from t_2_ to t_6_, corresponding to 40 min for an MC3T3 cell migrating across electrodes with FN coating alone. Consistent with optical measurements, the impedance magnitude signal duration was markedly longer for MC3T3 cells migrating on electrodes with APTES + FN coating, with average durations of 95.7 min and 298.0 min for FN and APTES + FN coatings, respectively, as shown in Figure 4b. The inverse relationship between cell migration speed and electrically measured duration of a cell passing electrodes validated the sensor’s utility for label-free tracking of single-cell motility.

In addition to the differences in impedance signal duration, single MC3T3 cells migrating on electrodes with APTES + FN coating generated significantly larger impedance magnitude changes than those on electrodes with only FN coating, as shown in Figure 4c. This enhancement in impedance magnitude was hypothesized to originate from variations in the cell–electrode contact distance. According to the established principles of ECIS, the measured impedance is highly sensitive to the nanometer-scale gap between the cell membrane and the electrode surface [44]. Because the cell membrane functions as an electrical insulator, ionic current has to flow through the narrow subcellular cleft before entering the electrolyte [14]. A smaller cell–substrate distance could constrain the current pathway and yield a higher impedance magnitude. To explore the biological basis for the reduced distance on APTES + FN-coated electrodes, the cell FAs were examined, which are complex macromolecular assemblies that physically link the intracellular actin cytoskeleton to ECM, maintaining a minimal distance of 10 to 15 nm between cell membrane and substrate [45,46]. An increased number or enlarged area of FAs could tighten the cell–substrate interface, restricting the current pathway beneath the cell and consequently amplifying the measured impedance signal.

To experimentally verify this mechanism, FA formation was investigated through immunofluorescence staining of MC3T3 cells cultured on glass substrates with FN or APTES + FN coatings, as shown in Figure 4d. Vinculin, a canonical protein component of FAs, was used as a marker and labeled as green. Cell nucleus was labeled as blue. The white triangles pointed to the FAs. The fluorescence images revealed that MC3T3 cells on the APTES + FN surface exhibited a larger number of FAs compared to cells on the FN-coated surface. This observation was substantiated by quantitative analysis shown in Figure 4e,f, which confirmed a statistically significant increase in both the number of FAs per cell and the total FA area per cell for cells on the platforms with APTES + FN coating. This enhanced adhesion likely reduced the effective cell–electrode gap, thereby explaining the larger impedance magnitudes recorded by the sensor.

Collectively, these results demonstrated that the developed device not only monitors the dynamic characteristics of single-cell migration but also provides insights into cell–substrate coverage and adhesion, highlighting its potential as a powerful tool for the integrated analysis of cell motility and morphological dynamics.

### 3.3. Comparative Impedance of MC3T3 and NP460 Cell Migration

Being able to distinguish different types of cells is particularly useful in biomedical research and diagnostics. Conventional techniques, such as flow cytometry, offer high-throughput quantification of cellular markers through fluorescence and light scattering. However, these methods typically require cell labeling, provide only snapshot measurements, and often necessitate cell detachment for adherent populations [47]. Traditional imaging-based migration assays could provide high-throughput monitoring of cell migration speed and morphology, but they typically require additional labeling to track cell adhesions on surfaces. In contrast, ECIS-based sensors enable label-free and continuous monitoring of adherent cells under physiological conditions, allowing for simultaneous tracking of adhesions, spreading, and migration. Building on the demonstrated sensitivity of the developed device to sense single-cell motility, adhesions, and spreading, its potential for label-free identification was further explored.

#### 3.3.1. MC3T3 Cells Induced Larger Phase and Real-Part Impedance Changes

**Figure 5:**
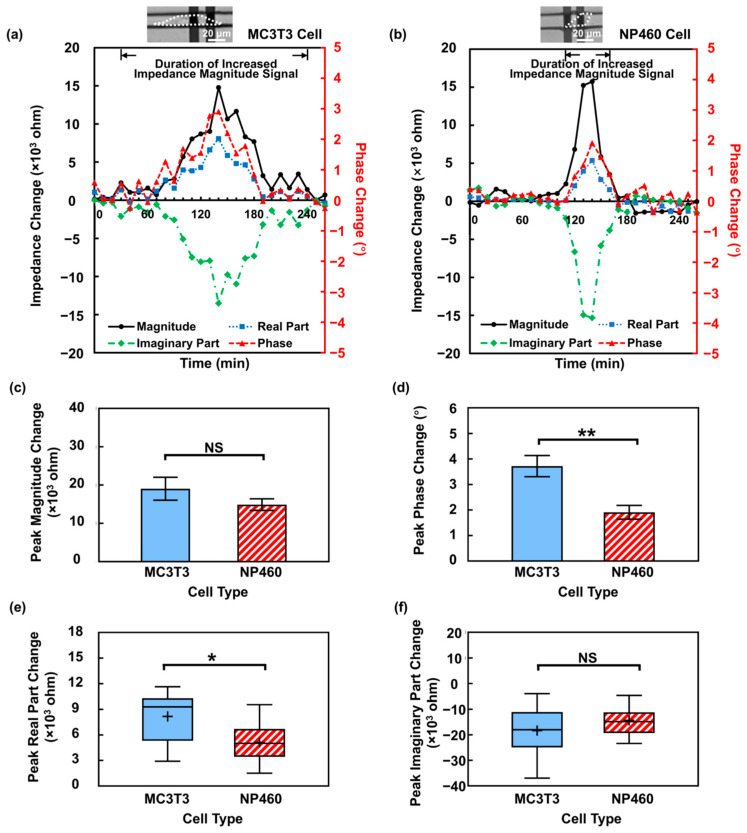
Impedance monitoring of MC3T3 and NP460 cells. Impedance and phase changes when (**a**) MC3T3 cell and (**b**) NP460 cell migrated across electrodes in channels. (**c**) MC3T3 and NP460 cells showed similar peak magnitude change. (**d**) MC3T3 cells induced higher peak phase change. (**e**) MC3T3 cells induced higher peak real-part change. (**f**) MC3T3 and NP460 cells showed similar peak imaginary part change. “+” represents mean value and horizontal line inside box represents median. One-way ANOVA and Tukey’s *post hoc* test with * *p* < 0.05, ** *p* < 0.01, and NS—not significant.

To this end, the sensor with APTES + FN coating was employed to detect MC3T3 and NP460 cells. Figure 5a,b show the impedance and phase responses of single MC3T3 and NP460 cells migrating on electrode pairs with APTES + FN coating. The time periods within the arrows indicate the durations of increased impedance magnitude signal, corresponding to the time ranges when individual cells migrated on electrodes as described above.

Quantitative analysis was performed to compare the impedance responses of these two cell types. The peak impedance magnitude changes were first compared, as shown in Figure 5c. Although MC3T3 cells showed slightly larger value, no significant difference in peak impedance magnitude changes was observed. In contrast, a significant difference appeared in the peak phase shift, as shown in Figure 5d. The MC3T3 cell induced an average peak phase change of 3.72°, whereas NP460 cells induced a change of 1.91°. To elucidate the origin of this phase difference, the impedance signals were decomposed into real and imaginary components, as shown in Figure 5e,f. The results revealed that MC3T3 cells produced greater changes in the real part of the impedance, while the imaginary part exhibited similar changes for both cell types.

**Figure 6:**
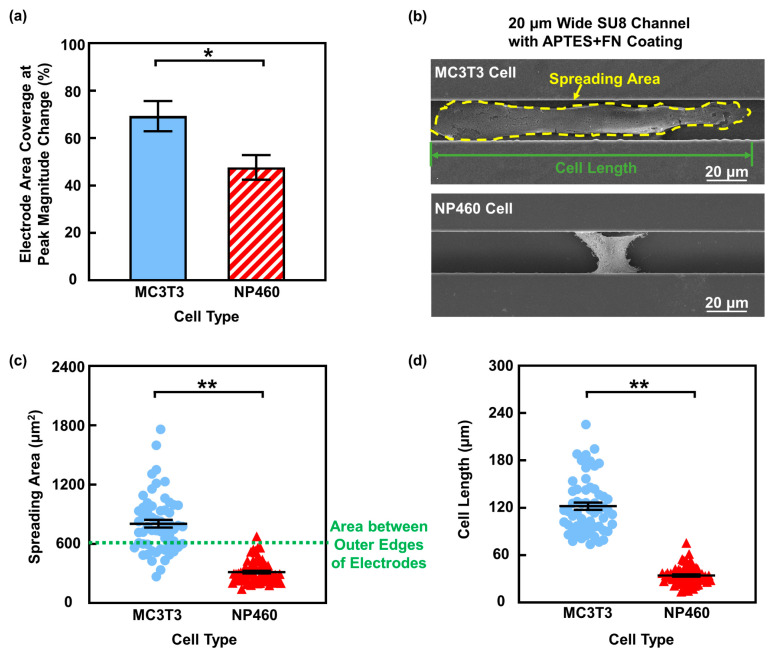
Electrode coverage and cell morphology in channels with APTES + FN coating. (**a**) MC3T3 cells showed larger electrode area coverage at peak impedance magnitude change. (**b**) Micrographs of MC3T3 and NP460 cells in channels. (**c**) MC3T3 cells had larger spreading area. (**d**) MC3T3 cells were longer than NP460 cells. One-way ANOVA and Tukey’s *post hoc* test with * *p* < 0.05 and ** *p* < 0.01.

The real component of impedance mainly represents resistive changes in the sensing circuit, reflecting the degree to which a cell restricts current flow when migrating over the electrodes. This blocking effect is closely related to the cell coverage and adhesion as discussed above. To clarify the reason for the larger impedance real-part changes observed for MC3T3 cells, the electrode area coverage was first analyzed. As shown in Figure 6a, MC3T3 cells showed a larger electrode area coverage of 69% at peak impedance magnitude change, while NP460 cell coverage was only 47%. This difference in electrode area coverage may arise from intrinsic morphological difference between the two cell types. To validate, SEM was used to visualize the morphology of MC3T3 and NP460 cells in 20 μm wide SU8 channels with APTES + FN coating, as shown in Figure 6b. The quantitative results in Figure 6c,d show that MC3T3 cells had larger spreading area and longer length along the channel direction compared to NP460 cells, enabling larger electrode area coverage and stronger resistive responses. In contrast, NP460 cells were smaller, with spreading area often less than the area between the outer edges of electrodes, resulting in partial electrode area coverage and weaker impedance changes. In addition to electrode area coverage, the cell adhesion characteristics on APTES + FN-coated surfaces were also examined. The immunofluorescence image of NP460 cells on glass with APTES + FN coating is shown in Supplementary Figure S4, revealing fewer and less prominent FAs compared to MC3T3 cells shown in Figure 4d. Reduced FA abundance and maturation in NP460 cells likely limit the cell–substrate coupling, which reduced the impedance real-part response. Together, the larger spreading area and more FAs in MC3T3 cells led to higher resistive changes observed.

#### 3.3.2. Rapid Movement of NP460 Cells Shortened Impedance Signal Change Duration

**Figure 7:**
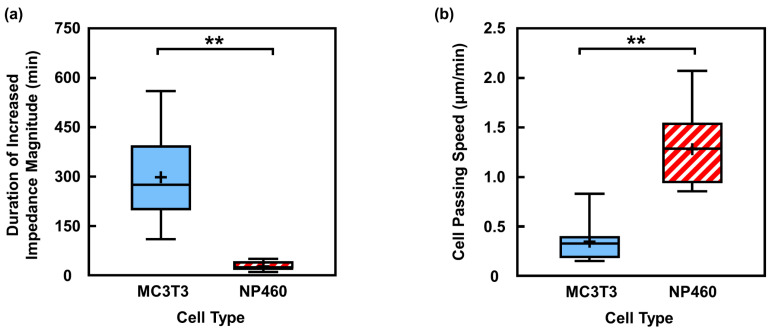
Comparison of increased impedance magnitude duration and passing speed when MC3T3 and NP460 cells migrated on electrode pairs with APTES + FN coating. (**a**) Duration of increased magnitude signal was longer for MC3T3 cells. (**b**) MC3T3 cells showed lower passing speed. “+” represents mean value and horizontal line inside box represents median. One-way ANOVA and Tukey’s *post hoc* test with ** *p* < 0.01.

In addition to morphological differences, the sensor was also capable of detecting variations in cell motility as demonstrated previously, providing an additional indicator for distinguishing cell types. The sensing length across the electrode pair was 30 µm, which required sustained cell migration to pass. As shown in Figure 7a, MC3T3 cells induced an average impedance signal increase duration of 298 min, while NP460 cells produced a signal increase duration of 31 min. These results indicated that the impedance signals reflected the cell migration passages across the electrodes. The longer signal increase duration observed for MC3T3 cells suggested a lower migration speed compared to NP460 cells. This observation was validated by quantifying cell passing speed from time-lapse imaging. As shown in Figure 7b, NP460 cells migrated faster than MC3T3 cells when passing electrodes with APTES + FN coating. Taken together, these results demonstrated that the impedance-derived parameters, including magnitude, phase, and signal duration, can collectively capture the morphological, adhesive, and motile characteristics of individual cells.

## 4. Discussion and Conclusions

In this work, an impedance sensor with electrode pair and protein-coated channel for monitoring adherent cell migration at single-cell level was developed and demonstrated. The electrode dimensions were optimized to maximize detection sensitivity. The proposed sensor effectively detected single-cell migration, revealing relationships between impedance signals and cellular motility, morphology, and adhesion. The different impedance signals induced by MC3T3 and NP460 cells highlighted its potential for label-free cell identification.

To enhance sensor performance, the effects of electrode gap and width on detection sensitivity were systematically characterized using the microfluidic device. A narrow electrode gap of 10 µm was found to be effective for achieving a large impedance magnitude change during single-floating-cell detection. For the electrode width, 10 µm yielded a slightly higher impedance magnitude change than 15 or 20 µm. Based on these results, both electrode gap and width were set to 10 µm, because this configuration produced the highest impedance magnitude change for single-cell detection. With the optimized electrode dimensions of 10 µm gap and 10 µm width, a substantial increase in impedance magnitude was observed when single MC3T3 cells migrated across the electrode pairs. Greater electrode coverage by the cell generally led to stronger current constriction and, thus, a larger impedance response. However, the relationship was not strictly linear for two reasons. Firstly, the electric field was not uniformly distributed over the electrode area, and the current density was typically higher near the electrode edges. A cell covering the electrode edges may, therefore, produce a larger impedance change than one covering other regions of equal area. Secondly, the impedance magnitude was also sensitive to the distance between the cell membrane and the electrode surface. For a given coverage area, a smaller cell–electrode gap led to a larger impedance magnitude change.

The sensor’s ability to detect cell behavior changes in different microenvironments was explored by modulating the surface protein coating. The duration of impedance signals was inversely proportional to migration speed. On APTES + FN-coated surfaces, MC3T3 cells migrated more slowly and produced longer duration of impedance signal changes compared to those on FN-coated surfaces. These results suggest that APTES + FN coating may lead to stronger cell–substrate coupling. When migrating cells attached more tightly to the underlying surface through mature FAs, their migration speed tended to decrease as stronger anchoring could hinder rapid cell detachment and forward movement. Meanwhile, stronger cell–substrate coupling led to reduced distance between the cell membrane and the electrode surface, resulting in a larger impedance magnitude. This was consistent with the observation that MC3T3 cells on APTES + FN-coated surfaces generated larger impedance magnitude changes than those on FN-coated surfaces. Quantitative analysis of FAs in cells supported this interpretation. On APTES + FN-coated surfaces, MC3T3 cells showed larger number and area of FAs. The increased FA number and area correlated with the tighter cell–substrate coupling on APTES + FN-coated surfaces, which reduced cell–electrode gap and, consequently, led to higher impedance signal changes observed on APTES + FN-coated surfaces. These results demonstrated that impedance sensing enables label-free and real-time monitoring of single-cell motility and adhesions without the need for continuous optical imaging or image processing. Several label-free techniques have been developed for single-cell migration and adhesion analysis. For example, holographic microscopy captures cell migration and morphology through amplitude and phase imaging. Resonant waveguide grating biosensing detects cell attachment via optical resonance shifts [48]. FluidFM directly measures single-cell adhesion forces through mechanical contact [49]. Each of these methods is grounded in distinct physical principles and captures specific aspects of cell behaviors. The impedance sensor developed in this work operates on a different principle based on cell electrical properties. It simultaneously records integrated electrical responses arising from cell adhesions, morphology, and motility, which serves as a complementary tool that introduces an electrical perspective to single-cell analysis.

The sensor was further investigated for its potential in label-free cell classification under identical experimental setups. With the fixed electrode configuration, channel dimensions, and protein coating conditions, variations in impedance responses among different cell lines primarily reflect intrinsic cellular properties rather than experimental variability. Comparative measurements of MC3T3 and NP460 cells revealed that impedance magnitude alone was insufficient to reliably distinguish different cell types. However, the consideration of multiple impedance-derived parameters, including changes in the impedance real component, phase shift, and signal change duration, produced distinctive electrical responses between two cell types under identical measurement conditions. MC3T3 cells, characterized by a larger spreading area and more FAs on APTES + FN-coated microenvironment, induced larger changes in impedance real part and phase with longer signal change durations. In contrast, NP460 cells exhibited faster migration speed and shorter duration of impedance changes.

Collectively, these findings indicate that the developed impedance sensor provides a label-free platform for the integrated analysis of single-cell motility, adhesion, and morphology. A single operating frequency 100 kHz was selected from the 10 to 100 kHz frequency range due to its lowest noise level. Despite the use of a single frequency, the measured electrical parameters including magnitude, phase, real, and imaginary components were sufficient to distinguish the behaviors of MC3T3 and NP460 cells under controlled conditions, highlighting the sensor’s sensitivity for monitoring single-cell migration speed and its potential for cell-type identification. While this study demonstrated differences between two cell types, the underlying principle can be extended to differentiate a broader range of cell types. To improve interpretability and enable a more comprehensive characterization of single-cell migration dynamics, future developments could incorporate multi-frequency impedance spectroscopy. This approach would allow extraction of additional biophysical parameters, such as cell membrane capacitance and gap resistance, helping to disentangle the relative contributions of cell spreading and adhesions to the recorded impedance signals. Furthermore, to improve the device’s working efficiency and address the inherent heterogeneity of cell populations, parallelization and cell positioning strategies can be implemented without altering the sensing principle. In this study, two electrode pairs in channels were demonstrated. The number of electrode pairs is scalable by using arrays of electrode pairs in parallel. This allows simultaneous measurements of impedance signals from a large number of single cells and is desirable for concurrent recording of many cells. Secondly, the throughput of single-cell detection can be improved by controlling cell passage through the channels. With optimized channel dimensions, guiding topography, and flow control in the channels, larger number of single cells will pass through the electrode pairs, resulting in improved detection efficiency. Additionally, the reusability of the impedance sensor is essential for reducing assay cost and improving working efficiency. After each run, cell debris and proteins secreted by the migrating cells in the channels may alter the surface properties of electrodes. A cleaning protocol can be used to render the device reusable. After each migration assay, the channels can be washed with trypsin solution to detach residual cells and secreted proteins, followed by rinsing with DI water and ethanol. Subsequently, an O_2_ plasma treatment can be applied to clean the surfaces of electrodes and SU8 channels. With proper cleaning and surface treatment, these impedance sensors can be reused for multiple runs. Overall, this methodology represents a step towards automated analysis of single-cell dynamics, streamlining the characterization of cell migration, and complementing traditional imaging-based assays.

## Data Availability

The original contributions presented in this study are included in the article/Supplementary Material. Further inquiries can be directed to the corresponding author.

## Supplementary Materials

The following supporting information can be downloaded at https://www.mdpi.com/article/10.3390/bios16050290/s1, Figure S1: Microfluidic device with single electrode pair for detecting floating cells passing through channels. (a) Fabrication technology for microfluidic device. (b) Micrograph of assembled device with 20 μm wide, 40 μm gap electrodes and 20 μm wide channel; Figure S2: Impedance magnitude of two NP460 cells migrating sequentially across pair of electrodes at (a) 10 kHz, (b) 33 kHz, and (c) 100 kHz. Red dashed line indicated noise level, which was defined as one standard deviation of the impedance magnitude value when no cell migrated on electrodes. Yellow shaded areas indicated periods when cell migrated on top of electrodes. (d) Time-lapse images of two NP460 cells migrating across pair of electrodes sequentially. Cell body is outlined by white dashes; Figure S3: Normalized impedance magnitude changes as single floating cells passing through channels when electrodes had gaps of 10 μm and widths of (a) 10, (b) 15, and (c) 20 μm. Channels were 20 μm wide; Figure S4: Immunofluorescence image of NP460 cell on glass with APTES + FN coating. Cell was stained to observe nucleus (blue) and vinculin (green).
