# Supplementary material for: Impedance Sensing and Characterization of Single-Cell Migration in Channels with Selective Protein Coating
Source: Biosensors (Basel). 2026 May 16;16(5):290. doi: 10.3390/bios16050290 (PMC13204719; doi:10.3390/bios16050290)
Supplement: Supplementary file 1 [file biosensors-16-00290-s001.zip › biosensors-4261490-supplementary.pdf]

Electronic Supplementary Information (ESI)

Impedance Sensing and Characterization of Single Cell Migration in Channels with Selective Protein Coating

X. Hong and S. W. Pang*
Department of Electrical Engineering
Centre for Biosystems, Neuroscience, and Nanotechnology
State Key Laboratory of Terahertz and Millimeter Waves
City University of Hong Kong, Hong Kong, China

*Corresponding Author:
S. W. Pang (pang@cityu.edu.hk)
Department of Electrical Engineering
City University of Hong Kong
83 Tat Chee Avenue, Kowloon
Hong Kong
Phone: +852 3442 9853
Fax: +852 3442 0562

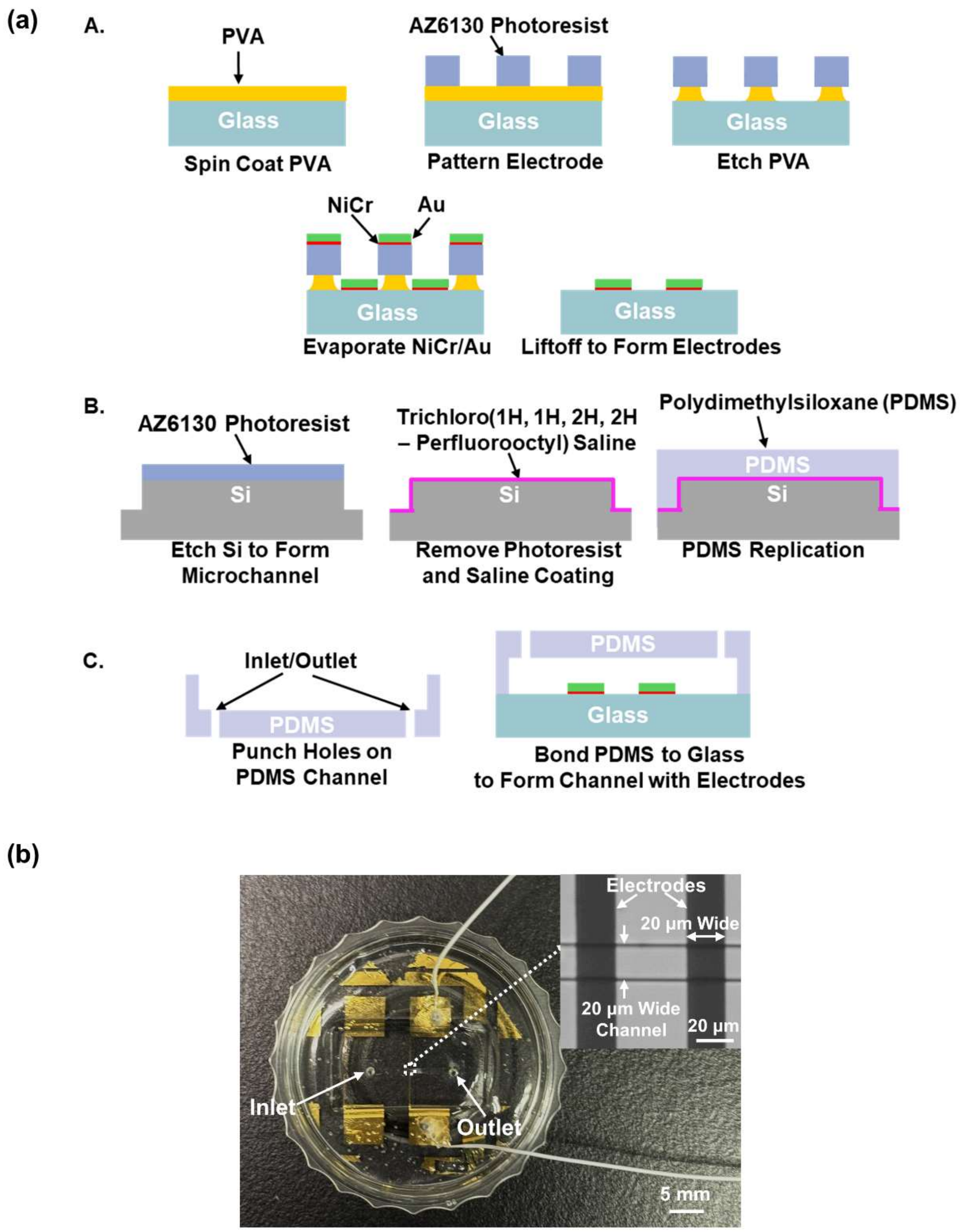

Supplementary Fig. S1 Microfluidic device with single electrode pair for detecting floating cells passing through channels. (a) Fabrication technology for microfluidic device. (b) Micrograph of assembled device with 20 µm wide, 40 µm gap electrodes and 20 µm wide channel.

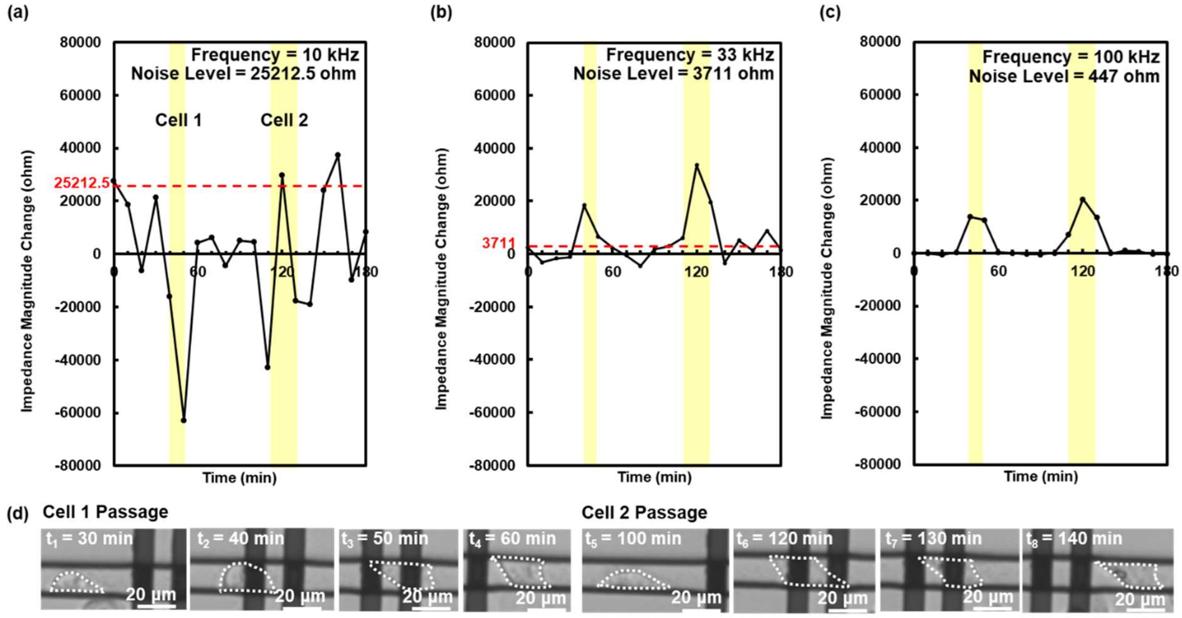

Supplementary Fig. S2 Impedance magnitude of two NP460 cells migrating sequentially across pair of electrodes at (a) 10 kHz, (b) 33 kHz, and (c) 100 kHz. Red dashed line indicated noise level, which was defined as one standard deviation of the impedance magnitude value when no cell migrated on electrodes. Yellow shaded areas indicated periods when cell migrated on top of electrodes. (d) Time-lapse images of two NP460 cells migrating across pair of electrodes sequentially. Cell body was outlined by white dashed line.

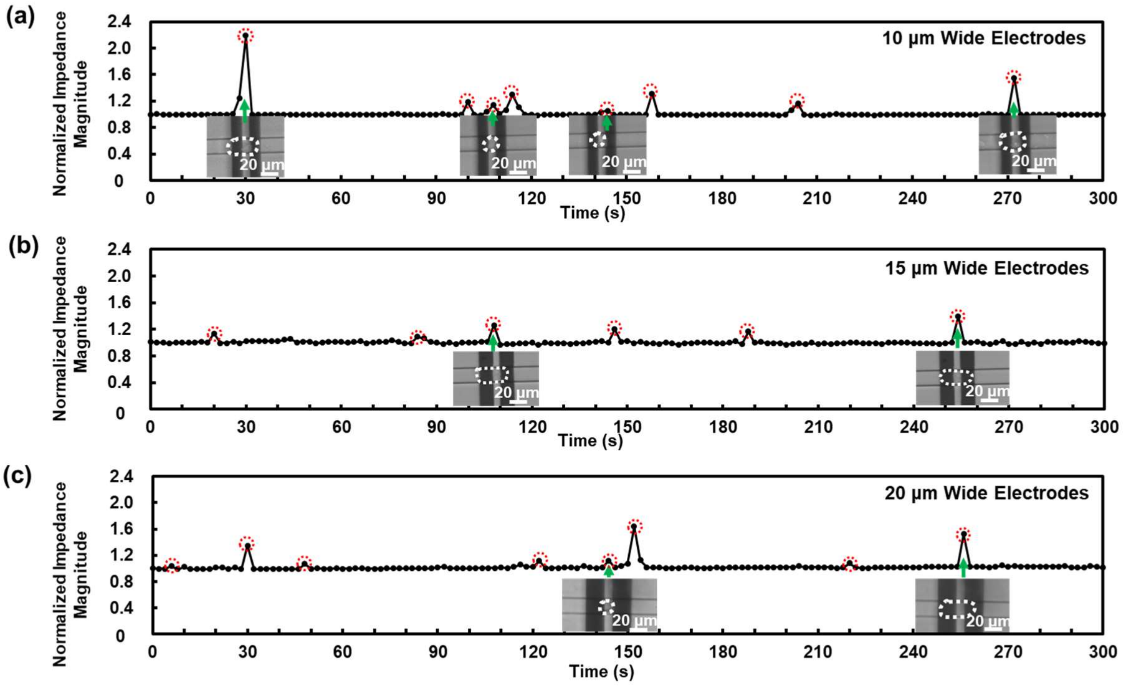

Supplementary Fig. S3 Normalized impedance magnitude changes as single floating cells passing through channels when electrodes had gaps of 10 µm and widths of (a) 10, (b) 15, and (c) 20 µm. Channels were 20 µm wide.

NP460 Cells

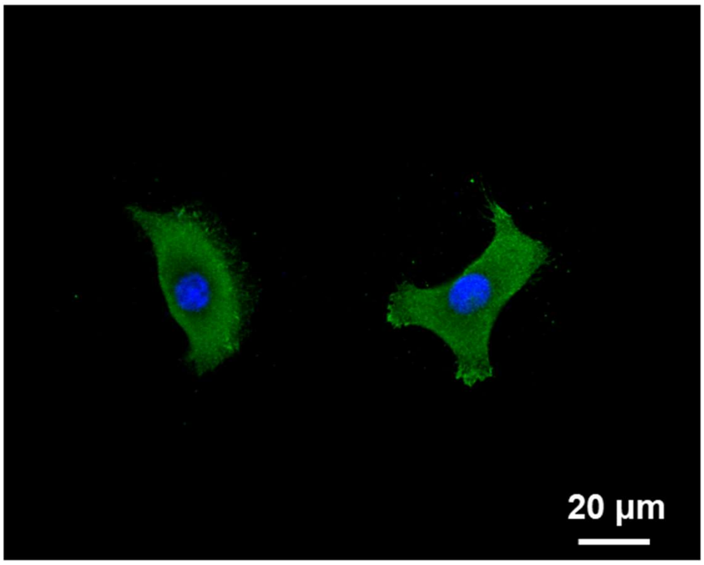

Blue : Nucleus
Green : Vinculin

Supplementary Fig. S4 Immunofluorescence image of NP460 cell on glass with APTES+FN coating. Cell was stained to observe nucleus (blue) and vinculin (green).
